# Formation of droplet interface bilayers in a Teflon tube

**DOI:** 10.1038/srep34355

**Published:** 2016-09-29

**Authors:** Edmond Walsh, Alexander Feuerborn, Peter R. Cook

**Affiliations:** 1 Osney Thermo-Fluids Laboratory, Department of Engineering Science, University of Oxford, Southwell Building, Osney Mead, Oxford OX2 0ES, UK; 2 Sir William Dunn School of Pathology, University of Oxford, South Parks Road, Oxford OX1 3RE, UK

## Abstract

Droplet-interface bilayers (DIBs) have applications in disciplines ranging from biology to computing. We present a method for forming them manually using a Teflon tube attached to a syringe pump; this method is simple enough it should be accessible to those without expertise in microfluidics. It exploits the properties of interfaces between three immiscible liquids, and uses fluid flow through the tube to pack together drops coated with lipid monolayers to create bilayers at points of contact. It is used to create functional nanopores in DIBs composed of phosphocholine using the protein α-hemolysin (αHL), to demonstrate osmotically-driven mass transfer of fluid across surfactant-based DIBs, and to create arrays of DIBs. The approach is scalable, and thousands of DIBs can be prepared using a robot in one hour; therefore, it is feasible to use it for high throughput applications.

Artificial bilayers, formed using the hydrophilic/hydrophobic properties of amphiphiles, have many applications in science. In addition to their main use in preparing “cell” membranes^1^,^2^,^3^,^4^, they have–for example–been incorporated into analogs of electrical circuits^5^ and next-generation DNA sequencers^6^, drug delivery methods^7^, used to facilitate diffusion between drops^8^ and in the assembly of individual drops into larger structures^9^. While planar lipid bilayers have been studied for decades in cell biology^10^,^11^,^12^, there is a growing interest in droplet interface bilayers (DIBs) formed by bringing together two drops coated with a monolayer^13^,^14^. Consequently, technologies to create DIBs continue to be developed, especially when DIBs are claimed to have advantages over their planar counterparts of higher temporal and mechanical stability, and no requirement for solid supporting structures; they can also easily be adapted to include asymmetric bilayers, and have the potential to be integrated into low-cost screening platforms^15^.

DIBs are typically formed using an appropriate amphiphile (e.g. surfactant or phospholipid) that forms a monolayer around a water drop. Such drops can then be organized into 1-, 2- or 3-D arrays using micromanipulators^1^, solvent extraction^16^ through polydimethylsiloxane (PDMS) and a variety of microfluidic devices^4^,^17^, since the surrounding monolayer prevents drops from fusing; then, bilayers form wherever two drops abut. Magnetic particles^18^ and electrowetting^19^ (in the context of digital microfluidics)^20^ have also been used to facilitate the formation of DIBs using lipids. DIBs formed from surfactants have also been created from multicomponent emulsions^21^. Alternatively, single bilayers can be created by bringing monolayer-coated drops next to planar monolayers using a tip^22^,^23^,^24^,^25^ or gravity; the latter was developed for high-throughput applications^26^,^27^.

Complex 1-, 2-, and 3-D networks of hundreds to thousands of interconnected bilayers have also been created using capillaries of variable size and geometry^15^,^28^ with rectangular and circular cross sections using two fluid phases and packing drops to form connected DIBs. Asymmetric DIBs can also be generated using a “lipid in” approach (where amphiphiles are added to the aqueous drop)^29^, but the amphiphile is usually added to the bulk oil phase. Then, however, large quantities of expensive lipids can be required, simply because the bulk phase must fill the large volume of the channels/chambers in the fluidic device used and the creation of independent DIBs is more complicated. In addition, such microfluidic devices often require dedicated chips, plus complex ancillary equipment. In the dedicated chips, usually made from PDMS, the choice of solvent for the lipids is also restricted due to their interaction with the substrate. For example, in an investigation on DIB formation the solvent hexadecane was used to reduce the swelling of PDMS^30^; and hence the range of experimental parameters may be limited^31^ due to solvent-substrate compatibility.

Herein, we describe an approach for making DIBs. It is simple enough to be implemented by any lab without expertise in microfluidics; it is scalable, utilizes minimal quantities of the appropriate amphiphile and does not require complex ancillary equipment other than a syringe pump; although high-throughput applications necessitate use of a robot. The approach utilizes a re-usable microfluidic device–a Teflon tube attached to a syringe pump and at least three immiscible fluids. Interfacial tension is exploited to create appropriate fluidic architectures, and to manipulate drops within them. We illustrate the approach by making 1- and 2-D arrays of DIBs using minimal quantities of amphiphiles, and go on to monitor transfer through DIBs, both generally and locally through functional nanopores. The differences of our approach to others is the reduction in amphiphiles required, the use of Polytetrafluoroethylene (PTFE) and fluorocarbon provides a much wider range of possible solvents which may be used, and each set of DIBs can be contained within a larger oil drop and hence independent experiments can be prepared within a single tube using greatly reduced quantities of amphiphiles. The tube approach also adds the benefit of reduced cost and simplicity compared with chip based devices.

## Methods

**Figure 1:**
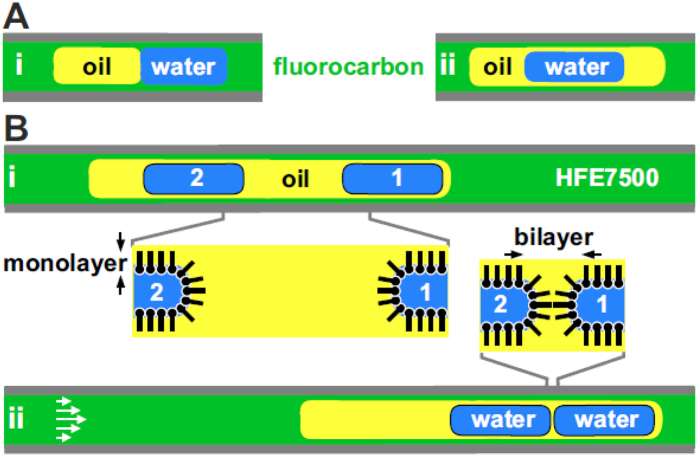
The approach. Three immiscible fluids are contained in a Teflon tube, and fluorocarbon always “wets” the Teflon. (**A**) Two fluidic architectures. (i) The three fluids can be stably in contact. (ii) Use of fluids with different interfacial tensions yields a different (stable) structure where oil engulfs water. This architecture is used throughout. (**B**) Creating DIBs (insets show structures of amphiphiles at oil-water interfaces). (i) The tube contains one super-drop of oil (a “train”) that engulfs two water drops; the oil contains an amphiphile that forms a monolayer at the oil-water interface (solid black line). (ii) During laminar flow (white arrows illustrate parabolic velocity profile), relative velocities are water drops >oil super-drop >bulk fluorocarbon (e.g., HFE7500) due to the liquid films between each fluid. As drop 1 cannot travel faster than the front of the oil super-drop, drop 2 soon catches it up; a DIB forms at the point of contact.

Our approach uses at least three immiscible fluids in a Teflon tube attached to a syringe pump; different fluids are used for different purposes. Figure 1Ai illustrates a typical fluidic architecture that can result when a fluorocarbon (phase 1), water (phase 2), and an oil (phase 3) are contained in such a tube; the fluorocarbon “wets” the Teflon to create a continuous and protective film along the tube wall so that any water-soluble molecules are unlikely ever to touch (or adhere to) the wall. The architecture can be created simply by dipping the tip of a tube filled with fluorocarbon successively into immiscible oil, water, and fluorocarbon as the pump is started and stopped appropriately. If the “Neumann triangle” is satisfied (i.e., the interfacial tension, γ, between any two fluids is less than the combination of interfacial tensions between the others, and γ_1–2_ < γ_1–3_ + γ_2–3_), interfaces between all three immiscible fluids can be in equilibrium^32^,^33^.

An alternative fluidic architecture results if γ_1–2_ > γ_1–3_ + γ_2–3_; now, oil engulfs water to create a drop-within-a-drop (Fig. 1Aii). We describe such a structure as a “train” (the oil drop), which in this case contains one “carriage” (the water drop). Trains with more “carriages” engulfed in one oil super-drop are prepared by including extra dips into water and oil before the last into fluorocarbon. Previously, we used flow to merge carriages within such trains, mix their contents, and transfer fluids between them by utilising surfactants and conditions that did not result in stable DIBs^34^.

In Fig. 1Bi, an amphiphile is added to the oil so that each of the two water drops in the train is coated by a monolayer (indicated by black lines). Starting the pump now causes laminar flow–where velocities are lowest at the edge and highest at the centre-line. Consequently, drops lying closest to the centre ride on higher velocity regions of the flow profile, and relative mean velocities are: water > oil >fluorocarbon^34^, see supplementary for further details and Fig. S2. Therefore, water-drop 1 has the potential to move faster than the surrounding oil, but is unable to penetrate the oil/fluorocarbon interface due to the engulfment interfacial tension condition. However, drop 2 can move faster, and so catches up drop 1^34^; consequently, a bilayer forms where the two drops abut (Fig. 1Bii). Importantly, if used with the appropriate fluids/amphiphiles, this approach ensures that one monolayer docks against the other, and this facilitates bilayer formation.

### Fluids and materials

HFE7500 was from Acota, Abil^®^EM180 from Surfachem, and all other fluids/materials from Sigma Aldrich unless otherwise stated. Where used, aqueous drops contained water-soluble dyes (Allura Red, toluidine blue and haematein). Amphiphile concentrations are given on a weight-to-weight basis unless otherwise stated. PTFE tubing of varying internal diameters, ultramicrobore tubing with internal/external diameters of 100/400 μm & 150/400 μm; 400 μm internal diameter tubing to allow joining with the ultramicrobore tubing (inserting one into the other) and 480 μm tubing for images with a larger field of view. For the measurements of velocity of drops the tube diameter was accurately determined as described in the Supplementary Material and found to be 343 μm internal diameter. Tubing sizes were selected based on practical considerations and we did not notice any variation in the fluidic structures associated with tube diameter range considered herein. Throughout this paper reference to tube diameter refer to the internal diameter unless otherwise indicated. The tubes was attached to a Harvard PhD Ultra I/W) high-precision programmable syringe pump fitted with Hamilton 800/1700 series air-tight glass syringes ranging in volume from 10 to 100 μl. Blunt needles of appropriate gauge were used to attach the tubing to the syringes.

Pores in bilayers were demonstrated using a 150-μm tube using fluids; HFE7500, silicone oil AR20 +amphiphile 1.5 mg/ml 1,2-diphytanoyl-sn-glycero-3-phosphocholine from Avanti Polar Liquids, Inc. Aqueous drops contained PBS with/without 100 mM pyranine (Sigma Aldrich) with/without 6 μg/ml α-hemolysin from *Staphylococcus aureus* (Sigma Aldrich). The use of silicone oil (AR20), rather than tetradecane as in all other experiments described herein, is due to the common use of this fluid in the literature^1^ for demonstrating pores in DIBs.

### Imaging

All images were collected using a digital camera (Nikon D7100 DSLR) connected to an epi-fluorescent microscope (Olympus IX53; 1.25X, 4X, 10X, 20X objectives) with translation stage and overhead illuminator (Olympus IX3 with filters) for bright-field images and LED wavelength-specific sources (CoolLED) and appropriate filter for fluorescent images. Image processing, analysis and illustrations were prepared using spreadsheets and CorelDraw. For several images, the PTFE tube was immersed in water to improve contrast with the walls of the tube for recording images.

### Forming fluidic architectures

The fluids utilised were; carrier fluid HFE7500, aqueous fluids (with/without additive), and tetradecane or AR20 as the separating fluids with amphiphiles. These fluids were pipetted into separate wells of a 96 well plate. A syringe pump (Harvard Ultra) with syringe/s fitted was used to withdraw the required volume of each fluid into a PTFE tube. Gas in the tubing system is problematic as it is compressible and therefore when the syringe pump starts/stops the fluid motion in the tube does not start/stop as rapidly as when no gas is present. To reduce the possibility of bubble formation in the tube/connectors/syringe the fluorocarbon was degassed using sonication and used to completely fill the system before forming the fluid architectures. To reduce the risk of cavitation fluorocarbons with a low viscosity/partial pressure are preferred.

The syringe pumps were programmed to operate in “withdraw/stop mode” where the start/stop timing and volume to withdraw were defined. The tip of the tube was immersed in the desired fluid, and then the required amount was withdrawn into the tube at flow rates ranging from 0.005–0.3 ml/h; next the pump was stopped, the tip moved to the next well containing a different fluid, and withdrawal mode restarted. This sequence can be repeated or altered by dipping into any well sequence to form the desired fluidic architecture. For example to create the simplest “train” of two aqueous drops engulfed in a separating fluid required a dipping sequence in wells containing–fluorocarbon–aqueous–separating oil–aqueous–fluorocarbon fluid. Additional aqueous drops can be engulfed in one oil super-drop by including extra dips into water and separating oil before the last dip into fluorocarbon. The resultant fluid architectures were unaffected by stops/restarts of the flow in the tube. Repeating this process provides any number of independent fluid architectures as the fluorocarbon is immiscible with both the separating oil and aqueous drops. By joining a tube loaded with trains with a second larger-bore tube, further DIB architectures may be formed. Although the dipping between reservoirs in a 96-well plate was usually performed manually using rack and pinion mechanism, a “robot” (Z-400, CNC Step, Germany) with a three axis-positioning system was used to demonstrate the potential of automated high-throughput applications.

## Results and Discussion

### Linear arrays of DIBs

**Figure 2:**
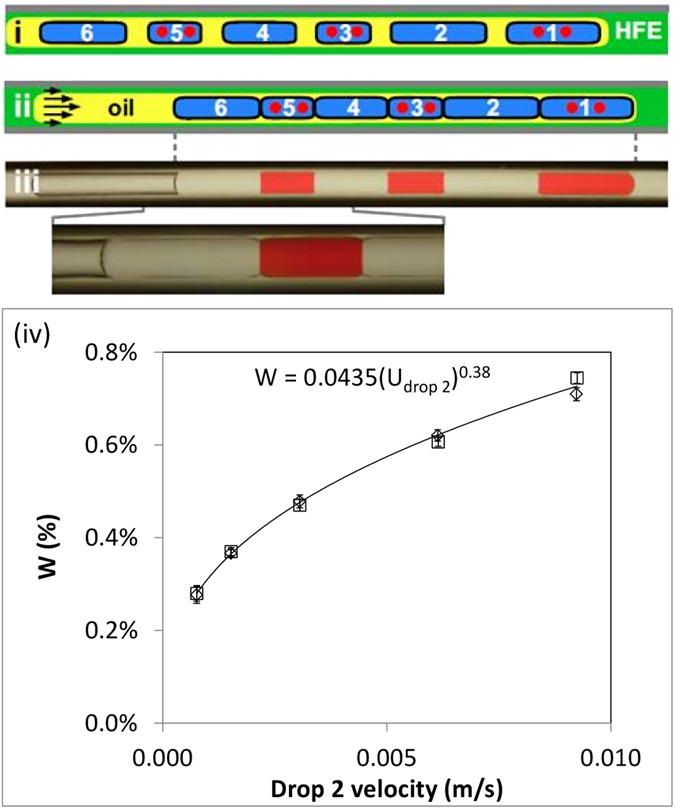
Forming a linear array of DIBs. (**i**) A train of 6 coated water drops (drops 1,3, and 5 contain red dye; 150-μm tube; fluids–HFE7500, water with/without red dye, tetradecane +3% AbilEM180). (**ii**) On flow, water drops travel faster than the oil to pack against each other; coating monolayers prevents drop fusion, and DIBs form where monolayers abut. (**iii**) Micrograph of the structure shown above (magnification below). (**iv**) The effect of flow rate on W, ratio of relative velocity between the first and second drops in the train of Fig. 1B using distilled water as the aqueous fluid in a 343 μm diameter PTFE tube. Each data point represents the average W from ten trains containing two drops, with error bars obtained from standard deviation. The two open symbol series represent independent repeat experiments.

In Fig. 2i, each of 6 aqueous drops in the train is coated by a monolayer, and every second water drop contains red dye. When laminar flow begins, water drop 1 cannot move faster than the interface, but drops 2–6 move through the oil to pack up against the preceding one (Fig. 2ii). The result is 6 “cells” separated by 5 DIBs at the interfaces, and each DIB has red dye on one side (Fig. 2iii). The area of each DIB is ~15,000 μm^2^, which is determined largely by the cross-sectional area of the 150-μm tube and ignoring the oil/carrier fluid film thicknesses that have widths much less than the tube radius^34^. The quantity of amphiphile-laden oil needed to create these DIBs is <20 nl (just that contained in the oil super-drop), and is at least one to two orders of magnitude less than that required with existing techniques that utilize channels or chambers filled with amphiphile^1^,^15^. Moreover, each water drop can have different compositions and so identical or asymmetric DIBs could separate different solutions. Additional independent trains can be created in the same tube that use different amphiphiles to give a set in which each individual DIB is different from the next. These features make the approach attractive for high-throughput screening and provide advantages over some existing related methods^15^. This novel method is similar in concept to the draining of fluid between individual aqueous drops as demonstrated using a PDMS microfluidic chip^30^, but herein this draining effect is achieved by fluid mechanics rather than forced by channel geometry. In addition this method is simplified over others (through the use of a single input port rather than multiple inputs) and uses the more chemical friendly PTFE rather than PDMS and thereby provides future scope for a wider choice of solvents.

It is important to consider where and when the DIBs will form in a tube for a given starting fluidic architecture; therefore the relative velocity difference between the first (labelled 1 in Fig. 1B) and subsequent drop/s (labelled 2 in Fig. 1B) as measured using an optical experimental technique (see Supplementary Information and Fig. S1) is shown in Fig. 2iv for the fluid combinations used herein. The prediction of the film thickness and hence velocity difference of the engulfed drops (see Supplementary Information and Fig. S2), has been extensively studied^28^,^30^,^31^,^32^,^33^,^34^,^35^,^36^ since the original experimental^35^ and theoretical works^36^; considering two immiscible fluids. Most theories/experimental data of this type identify the Capillary number [Ca = μV/γ] as the relevant scaling parameter in low Reynolds number flows. The best-fit power law to this data is


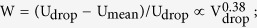


where U_mean_ and U_drop_ are the measured velocity of the first (equal to the oil super-drop velocity) and the second aqueous drops before they abut; which for constant viscosity and interfacial tension provides the same scaling with Capillary number. The resultant scaling is in broad agreement with existing data for two and three phase droplet systems using different fluids and surfactants^35^,^34^.

This relationship between relative droplet velocity within a single train may then be used to predict when and where in the tube the DIBs will form. For example, the velocity difference between the first and second aqueous drop in an oil super-drop with a mean velocity of 2 mm/s is 8 μm/s (i.e. W = 0.4%). Therefore, for an initial spacing of 0.5 mm between the first and second drops at the inlet of the tube; a DIB would be formed between these drops after ~62.5 seconds at a distance of ~125 mm from the inlet of the tube.

The stability of DIBs formation was recently quantified using a PDMS microfluidic device^30^, by considering the velocities entering a “shift register” to create DIBs. It was found for the range of amphiphile concentrations from 2–10 mg/ml that the drop velocity could be increased from ~50 to 200 μm/s, respectively, and result in stable DIBs. For DIB experiments herein the relative velocities between drops was a maximum of ~10 μm/s and found to be stable with the fluids used, HFE-7500, tetradecane +3% Abil EM180 and aqueous solutions.

### DIBs pierced by nanopores

**Figure 3:**
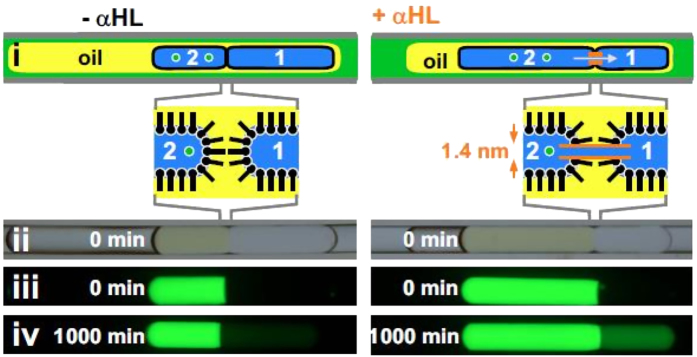
αHL nanopores (150-μm tube; fluids–HFE7500, PBS with/without αHL, silicone oil AR20+ phosphocholine (1.5 mg/ml)). Drop 2 contains (green fluorescent) pyranine (100 mM). (**i**) Flow has packed coated drops 1 and 2 together to create a DIB between the two (insets). If present, αHL forms nanopores (right) which enhances the rate of pyranine transfer (arrow). (**ii**) Bright-field image of the structure shown above: drops 1 and 2 abut. (**iii,iv**) Fluorescence images: after 1000 min αHL has facilitated a much higher transfer of pyranine from drop 2 to 1.

We next created “cells” connected by nanopores using DPhPC, and addition of monomers of the bacterial toxin–α-hemolysin (αHL)–can lead to spontaneous assembly of heptameric 1.4-nm pores in such bilayers^1^,^4^,^16^. Although this protein/pore is a relatively simple structure and others may be of more interest for screening purposes, it is used here for proof of concept purposes as it is the most widely used in the microfluidic literature. These bilayers are easily made using an oil drop engulfing water drops when if the oil is saturated with phosphocholine; then, flow packs one coated drop against another, and a DIB forms between the two (Fig. 3i,ii, left). In the absence of αHL, if the second drop contains the fluorescent dye, pyranine, minimal dye diffuses into the first over 1,000 minutes (as the DIB is largely impermeable, and the oil largely immiscible to the dye; Fig. 3iii,iv, left). However, if both drops initially contain αHL, nanopores assemble in the bilayer, and the dye can now diffuse from one drop to the other at an accelerated rate, facilitated by the nanopores created by αHL (Fig. 3, right), noting that the fluorescence level was detectable in <50 minutes. If the two drops containing αHL are brought close together without touching, so bilayers cannot form, there is no measurable transfer of dye between drops through the oil phase over 3 days (Fig. S3).

### Osmotically-driven transport of water across DIBs

**Figure 4:**
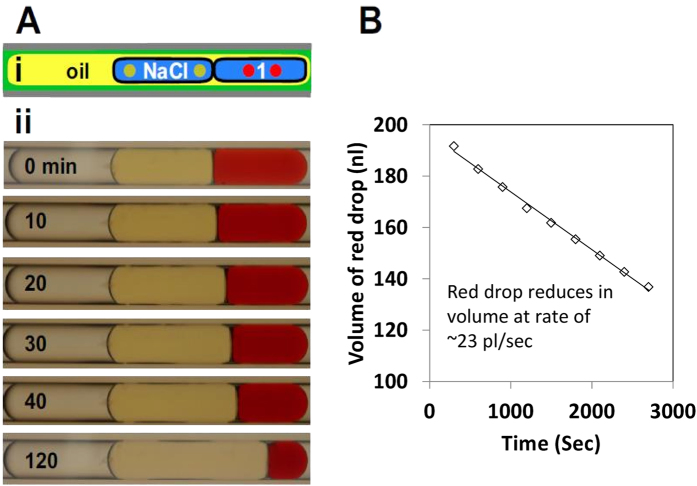
Osmotically-driven fluid transfer across a DIB. (**A**) Cartoon and images. (i) Fluidic architecture in a 460-μm tube with fluids–HFE7500, tetradecane +3% EM180, and 5M NaCl water or water +3 mg/ml Allura red, traversed at a velocity of ~1.1 mm/s, giving an excess velocity of the 5M NaCl drop over the red drop of ~3.5 μm/s calculated from Fig. 2 (iv). A DIB forms between the first water drop (containing red dye), and the second drops; both water drops initially have volumes of ~200 nl. (ii) Bright-field images of the tube shown above; over time, osmosis drives fluid from drop 1. (**B**) Volume of the red drop at different times obtained from images on left; repeated experiments found an approximately constant diffusion rate in the initial phase (linear slope in curve fit) with a variation of ~20%.

Water and small molecules can be transferred through DIBs in a microfluidic chip by concentration gradients^8^,^37^. In our system, an analogous water transfer is achieved using a surfactant-based DIB between two drops in a tube; the first contains red dye, the second 5M NaCl (Fig. 4Ai). Over the next ~1 hour, the red drop shrinks as the other enlarges (Fig. 4Aii); at a rate of 23 pl/s (Fig. 4B), and this transfer rate is reduced by over three orders of magnitude when the two drops are brought close together without abutting (Fig. S4). This approach provides a simple way of studying mass transfer through DIBs. It could also be used to concentrate proteins and precipitant solutions when screening conditions to generate protein crystals; this method being analogous to the classical methods that utilize vapour diffusion. To achieve a similar result the permeability of a PDMS wall has been exploited to enable osmotic driven flow^38^, while here the same effect is achieve in a simple tube. This method could be used, for example, to measure the permeability coefficient of lipid bilayers as studied by others using spherical drop pairs^31^ or large arrays of drops^37^; but has the advantage of a well-defined bilayer interface area and easily measured volume change of drops.

### Arrays of DIBs

**Figure 5:**
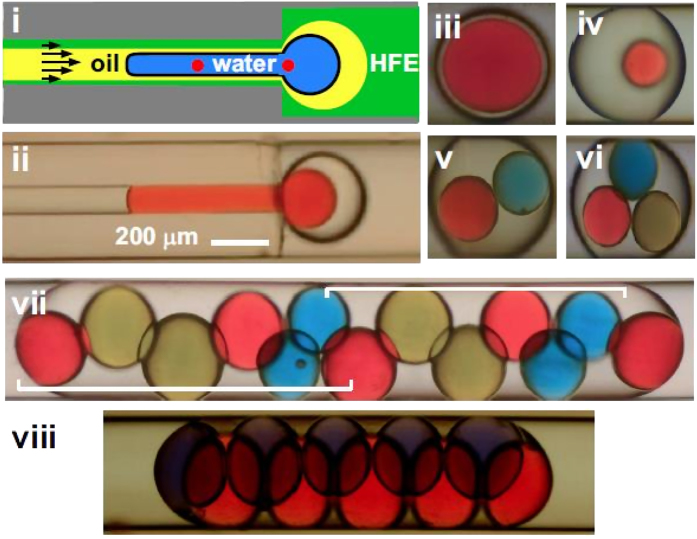
Arrays of DIBs (fluids–HFE7500, water + red/blue/yellow dye, tetradecane +3% AbilEM180). (**i**) An oil super-drop engulfs a coated water-drop as it flows from one tube (100-/400-μm internal/outer diameter) to another (400-μm internal diameter). (**ii**) Still from movie of the structure shown above. (**iii,iv**) Micrographs of single coated water-drops within one oil drop deposited in the wider tube; no DIBs are present. (**v,vi**) DIBs form when coated drops are in contact. (**vii**) An ordered array of coated drops and DIBs (brackets indicate repeating pattern). (**viii**) A tightly-packed array in which each of the 6 central drops form DIBs with 4 others.

More complex arrays of DIBs can be prepared using tubes with different diameters. In Fig. 5i,ii, tubes with 100- and 400-μm bores have been connected, and a coated water-drop is passing from one to the other. Once the engulfing oil drop has left the small tube, it forms a sphere as long as the diameter is small enough to fit in the larger tube. Then a spherical water drop is engulfed in a larger spherical, oil drop. The relative volumes of oil and water can easily be varied by drawing different amounts into the tube at the beginning, and this allows double emulsions with different amounts of water and oil to be created (Fig. 5iii,iv). If the original train contained two (or three) water drops, the result is an oil drop containing two (or three) water drops. And if the volume of the oil drop is small enough to force the water drops together, DIBs form at points of contact (Fig. 5v,vi and Movie S1). Here, tight packing increases DIB area.

Double emulsions with any number of water drops are easily created using appropriate trains. For example, in Fig. 5vii there are 13 water drops containing dyes of different colours, and (after the leading red drop) the pattern blue, blue, red, yellow, yellow, red repeats (see also Movie S2). This result is obtained because of geometric constraints; the diameter of each water drop is greater than half the diameter of the larger tube, so drops cannot pass preceding ones and hence drops maintain their original positions in the train. Figure 5viii illustrates a structured array of 10 drops and 17 DIBs, where each of the central 6 drops forms bilayers with 4 others. Here, the volume of the engulfing oil is reduced so aqueous drops are tightly packed together to increase the area of each DIB (see also Movie S3). Such structures are reminiscent of those made using many tubes^28^, but here packing is controlled by the third immiscible phase.

### High throughput

**Figure 6:**
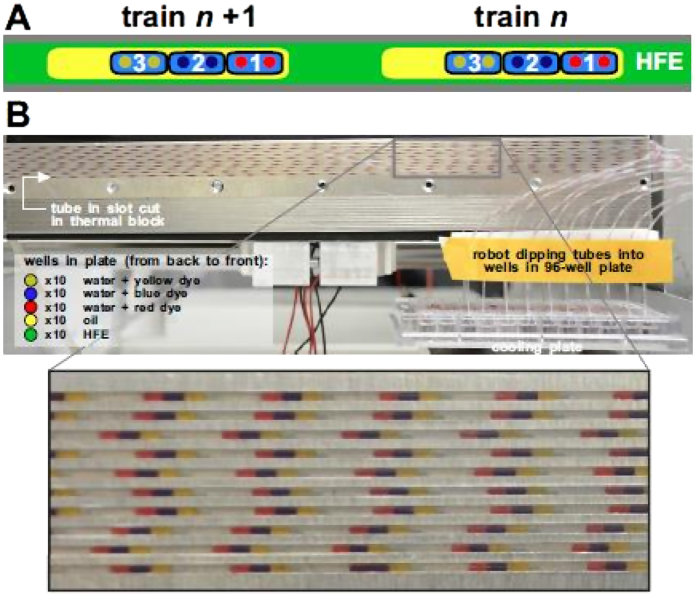
High-throughput generation of hundreds of DIBs. Ten 480-μm tubes are attached to 10 syringes driven by one pump; the other ends of the 10 tubes are dipped by a robot successively into appropriate fluids in a 96-well plate to generate a series of identical trains (each with three 200-nl water drops containing a dye of different colour). Flow packs drops coated with monolayers against each other to create 2 DIBs/train. (**A**) Fluidic architecture of two of the identical trains in series in one tube (fluids –HFE7500, water + red, blue or yellow dye, and tetradecane +3% EM180). (**B**) A still from Movie S4 (inset shows magnified region). The tubes are attached to syringes (out of sight) and they pass through an aluminium block with recessed channels of 300 mm long to hold the tubes. Their other ends are being dipped by the robot into the 96-well plate (bottom right; arrangement of wells indicated on the left). Series of trains with water drops of different colours can be seen in each tube (inset). The entire train array loaded into tubes with a continuous stop/start between dipping in the wells as shown in Movie S4.

Thus far, DIBs have been created within one train, and when there is more than one DIB/train, all DIBs are chemically similar (although each may form a barrier between different aqueous solutions). More, and potentially-different, DIBs between “cells” with different contents can be created using more trains in series and tubes in parallel. In Fig. 6 and Movie S4,10 tubes are attached to 10 syringes driven by one pump, and a robot dips tube ends into wells containing fluorocarbon, different aqueous solutions containing red, blue, or yellow dye, and tetradecane plus 3% EM180. The result is a series of trains, each with three water drops that carry differently-colored dyes (Fig. 6A). As each train forms, the amphiphile in the surrounding oil coats each water drop and flow induces the three now-coated drops to pack against each other; consequently two DIBs form in each train. As ~40 trains (each with two DIBs) are created per tube in ~20 min, over a thousand of the same or different DIBs (separating the same or different solutions) could be created in one hour using a single syringe pump.

## Conclusions

This paper details a simple microfluidic approach for creating DIBs using both lipid and surfactant-based amphiphiles. It exploits knowledge of interfacial tension and fluid mechanics, rather than the more classical approach of channel geometry, to create specified fluidic architectures in three immiscible fluids, and then to pack together drops coated with monolayers so that DIBs form at points of contact. The method is relatively simple, requiring only a single (reusable) tube and syringe pump, and it requires minimal quantities of (sometimes expensive) amphiphiles. The area of the DIB can be increased simply by increasing tube diameter. Once formed, DIBs are protected within the tube, and ones like those shown in Fig. 2 have been kept without change for 5 days. Arrays of varying complexity can be created, ranging from artificial “cells” connected by nanopores to more complex 2-D DIB architectures. Most of our results were obtained manually using a Teflon tube and syringe pump, so the method should be accessible to those without expertise in microfluidics. Finally, the approach is also scalable, so that–with the addition of a robot–thousands of independent DIBs can be prepared in one hour. As DIBs can be formed using different trains, it is then possible to perform high-throughput screens involving different kinds of DIB separating different kinds of aqueous compartment.

## Additional Information

**How to cite this article**: Walsh, E. *et al*. Formation of droplet interface bilayers in a Teflon tube. *Sci. Rep.* **6**, 34355; doi: 10.1038/srep34355 (2016).

## Supplementary Material

Supplementary Information

Supplementary Movie S1

Supplementary Movie S2

Supplementary Movie S3

Supplementary Movie S4
