# Supplementary material for: Formation of droplet interface bilayers in a Teflon tube
Source: Sci Rep. 2016 Sep 29;6:34355. doi: 10.1038/srep34355 (PMC5041184; doi:10.1038/srep34355)
Supplement: Supplementary Information [file srep34355-s1.pdf]

Supplementary Information

Formation of droplet interface bilayers in a Teflon tube

Edmond Walsh,[1]** Alexander Feuerborn*[2] *and Peter R. Cook*[2].

1. *Osney Thermo-Fluids Laboratory, Department of Engineering Science, University of Oxford, Osney Mead, Oxford OX2 0ES, UK. Email: edmond.walsh@eng.ox.ac.uk; Fax: +44 1865 288756; Tel: +44 1865 288731.

2. Sir William Dunn School of Pathology, University of Oxford, South Parks Road, Oxford OX1 3RE, UK.

Measurement of drop velocity and length

For Figure 1C; drop velocity was measured as detailed previously[32] using two orthogonal LEDs/photodiodes spaced 1 m apart along a transparent PTFE tube (343 μm bore; photodiodes were ~1.3 m from tube ends; Fig. S1). As all fluids used in a single experiment had different refractive indices, photodiode voltage varied depending on the fluid in the light path. Times taken by drops to travel through one light beam and between beams were recorded using custom software, with sampling frequency of up to 500 Hz and thereby provide negligible error of the average velocity of drops over the 1 m distance between photodiodes.

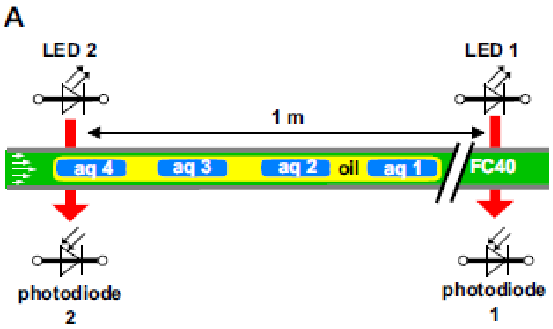

Fig. S1. Experimental setup for the measurement of drop length and velocity; photo-diode voltage reflects which fluid is in the light beam. Times taken by drops to travel between beams were recorded and velocity of each oil and water drop calculated.

It is important to determine tube diameter accurately since the film regions are very thin, so HFE7500 was pumped through a virgin tube at a known flow rate. The time taken for the leading HEF7500-air interface to travel between the two photodiodes was recorded, and average diameter calculated using the continuity equation and known flow rate set on the syringe pump. This must be done prior to any other fluids entering the tube as otherwise there may already be a film on the wall which would result in a lower tube diameter being calculated.

Drop velocity and film thickness

The thickness of a film engulfing a drop (as in Fig. 3) may be estimated by implementing the assumptions of an inviscid ($\mu_1 >> \mu_2$) or solid drop ($\mu_1 << \mu_2$), and applying continuity to the flow within a circular tube. For an inviscid drop, where *R* = channel radius, *r* = drop radius, *h* = film thickness, *Q* = flow rate, *U* = mean velocity of fluids, we obtain:

$$\dot{Q}_{mean} = \dot{Q}_{film} + \dot{Q}_{drop}$$

The velocity in the film region is modelled as zero or a linear velocity profile (Couette flow) to determine the limits of the film thickness for the cases of inviscid or solid drops respectively (Fig. 3ii). Equating flow rates at axial positions in carrier fluid (phase 1) and drop region (phase 2) yields:

$$\left[\pi R^2 U_{mean} = \pi (R-h)^2 U_{drop}\right]_{inviscid}$$

$$\left[\pi R^2 U_{mean} = \pi \left(R^2 - (R-h)^2\right)\frac{U_{drop}}{2} + \pi (R-h)^2 U_{drop}\right]_{Visc}$$

Solving these equations where $h << R$, provides

$$\left[\frac{h}{R}\right]_{\text{inviscid drop}} = \frac{1}{2}W$$

$$\left[\frac{h}{R}\right]_{\text{solid drop}} = \mathrm{W}$$

$$W = \frac{U_{drop} - U_{mean}}{U_{drop}}$$

These equations, or similar, have been derived by several authors for an inviscid[37,38] and solid drop.[32,39] Therefore, when flow is induced in the capillary illustrated in Fig. S2, the distance between x_f and x_d reduces by Δx which depends on film thickness. The film thickness, in the limits of an inviscid or solid drop, may be estimated by measuring the velocities of the drop and carrier fluid. For all cases where $\mu_1 \sim \mu_2$, it is expected to reside between these two limits. The same equations can then be applied to our new fluidic architecture illustrated in Figure 1A, where film thickness

between water and oil phases may be estimated by measuring the mean velocity of both (assuming the carrier-fluid film surrounding the oil is unchanged over the length of the oil drop). The resultant film thicknesses, from the inviscid and solid drop equations, provide the limits of film thicknesses for any viscosity ratio between water and the engulfing oil drops.

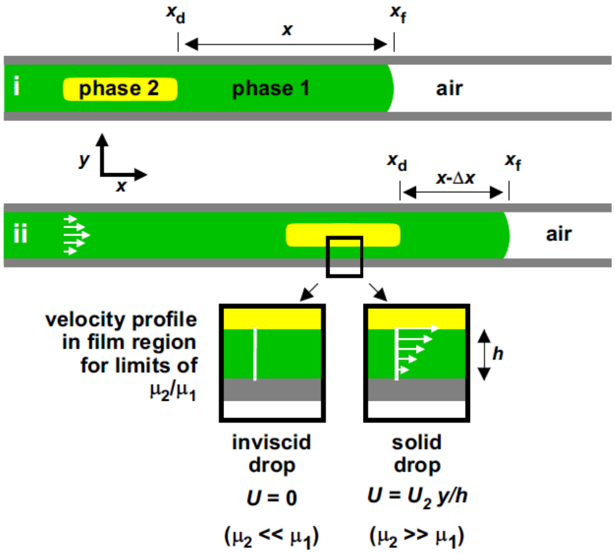

Fig. S2. Schematic of immiscible fluids flowing in a circular capillary; (i) phase 2 (with leading interface at x_d) is engulfed by phase 1 (with leading interface at the air, x_f), which wets the wall. (ii) After flow, the distance between interfaces x_f and x_d has reduced due to the velocity of phase 2 (which is > phase 1). Also illustrated are the simplified velocity profiles in the film region between phase 2 and the wall, for an inviscid and solid drop.

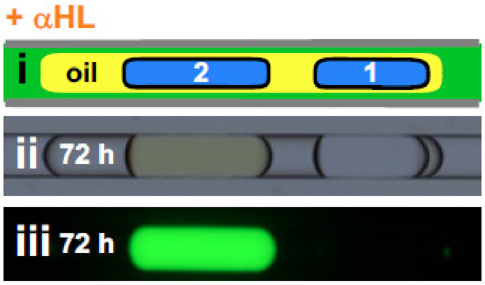

Fig. S3. Transfer through αHL nanopores depends on contact between coating monolayers. This is a control for the experiment illustrated in Fig. 3, where DIBs form when two water drops coated with monolayers are in contact (150-µm tube; fluids – HFE7500, PBS, silicone oil AR20 + phosphocholine). Conditions are the same as in Fig. 3, except that flow was stopped before water-drop 2 caught up 1; consequently, no bilayers (or nanopores) form. (i) Architecture. (ii) Bright-field image of the structure shown above: after 72 h, drops 1 and 2 are still not in contact (no flow occurs during the 72 h). (iii) Fluorescence image of the structure shown above: after 72 h, no fluorescence signal is detected in drop 1; this indicates that pyranine is unable to diffuse through the oil from drop 2 to 1, and confirms that the formation of functional pores in Fig. 3.

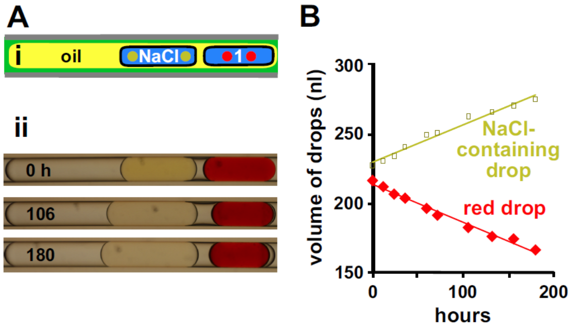

Fig. S4. Little osmotically-driven fluid transfer occurs between two coated drops if they are not in contact. This is a control for the experiment illustrated in Fig. 4. Conditions are the same as in Fig. 4, except that flow was stopped before water drop 2 caught up 1; consequently, no bilayer forms. (A) Cartoon and images. (i) Fluidic architecture (480-μm tube; fluids – HFE7500, tetradecane + 3% EM180, and water +3 mg/ml Allura red and 5M NaCl solution). No DIB forms, because the coated drops are not in contact; both water drops initially have volumes of ~200 nl. (ii) Bright-field images of the tube shown above; over 180 h, osmosis drives fluid from drop 1 to 2 through the oil. (B) Volume of the red and yellow drops at different times. The rate of transfer between drops 1 and 2 is ~3,000-fold slower than in Fig. 4 (where coated drops were in contact, and a DIB formed). Repeated experiments show variation in diffusion rates (slope of line) of ~40%, mostly due to controlling the distance between the first and second drop exactly.

Movies

Movie S1

2-D array of DIBs. This Movie illustrates the formation of the structure illustrated in Fig. 5vi. A train consisting of 3 coated water drops (containing yellow, blue, and red dyes) passes from the thin tube into the thick one. This results in the formation of 3 water drops engulfed in one oil drop; DIBs form at points where one coated water drop contacts another.

Movie S2

2-D array of DIBs. This Movie illustrates the formation of the structure illustrated in Fig. 5vii. A train consisting of 13 coated water drops (containing yellow, blue, or red dyes) passes from the thin tube into the thick one. This results in the formation of 13 water drops engulfed in one oil drop (water drops retain their original sequence); DIBs form at points where one coated water drop contacts another.

Movie S3

Compact array of DIBs. This Movie illustrates the formation of the structure illustrated in Fig. 5viii. A train consisting of 10 coated water drops (containing red dye) passes from the thin tube into the thick one. This results in the formation of 10 water drops engulfed in one oil drop. Water drops retain their original sequence, and are tightly packed; 17 DIBs form at points where one coated water drop contacts another (the 6 central drops form bilayers with 4 others).

Movie S4

High-throughput generation of hundreds of DIBs. This Movie illustrates Fig. 5. Ten tubes are attached to 10 syringes (out of sight at top left); they pass through a thermal block at the top (not used here), as their other ends are dipped by the robot into a 96-well plate on a cooling plate (also not used here). Series of trains with water drops of different colours can be seen in each tube. Flow gently packs coated drops against each other (to form DIBs) by the time trains reach the thermal block.
